# Trends in Post-Secondary Student Stress: A Pan-Canadian Study

**DOI:** 10.1177/07067437221111365

**Published:** 2022-07-06

**Authors:** Brooke Linden, Heather Stuart, Amy Ecclestone

**Affiliations:** 1 Department of Public Health Sciences, Health Services and Policy Research Institute, 4257 Queen’s University, Kingston, Ontario, Canada

**Keywords:** mental health, stress, post-secondary, well-being, COVID-19, higher education

## Abstract

**Objective:**

Previous research has evaluated the sources of post-secondary student stress, but has failed to explore whether stressors fluctuate over time. The purpose of this research was to use the Post-Secondary Student Stressors Index to examine whether stressors changed significantly and meaningfully over the course of an academic year. Due to the timing of data collection, results also provide context around students’ experiences of stress during the COVID-19 pandemic.

**Method:**

Cross-sectional data was collected at 3 time points via online surveys over the course of the 2020–2021 academic year from >10,000 students. Participants attended 15 post-secondary institutions across Canada, representing 9 provinces and 1 territory. Validated instruments were used to assess levels of stress, distress and the severity of student-specific stressors. Kruskal–Wallis ranked tests and multiple pairwise comparison analyses were conducted to assess whether the mean severity of stressors changed over time. Standard effect sizes were calculated using Cohen's *d*.

**Results:**

Mean levels of stress and psychological distress were high at the start of the study and remained high across time points. A similarly high level of stress was observed on average for student-specific stressors. While significant differences in mean severity were observed over time for some stressors, standardized effect sizes were negligible, suggesting little meaningful change and consistent levels of chronic stress over the course of the academic year.

**Conclusions:**

This is the first paper to examine trends in student-specific stress using a nationwide sample of Canadian post-secondary students during the first year of the COVID-19 pandemic. Patterns observed in student-specific stressors reflected changes likely to be indicative of the pandemic, including the most severe stress associated with academics, finances and concerns for the future. Implications for future research are discussed, in particular, the importance of examining stressors related to COVID-19 and their impact on student mental health.

## Abrègè

**Objectif:**

Une recherche précédente a évalué les sources de stress chez les étudiants de niveau post-secondaire, mais n’a pas exploré si les facteurs de stress fluctuaient avec le temps. Le but de la présente recherche était d’utiliser l'indice des facteurs de stress chez les étudiants de niveau post-secondaire afin d’examiner si les facteurs de stress changeaient significativement et de façon importante au cours d’une année scolaire. Étant donné le moment où les données ont été recueillies, les résultats procurent aussi un contexte aux expériences de stress des étudiants durant la pandémie de la COVID-19.

**Méthode:**

Les données transversales ont été recueillies à trois moments différents par des sondages en ligne au cours de l’année scolaire 2020–-2021 de la part de >10 000 étudiants. Les participants fréquentaient 15 institutions post-secondaires du Canada, représentant neuf provinces et un territoire. Des instruments validés ont servi à évaluer les niveaux de stress, la détresse et la gravité des facteurs de stress spécifiques des étudiants. Le test de Kruskal-Wallis a servi à coter les tests et de multiples analyses de comparaison par paires ont été menées pour évaluer si la gravité moyenne des facteurs de stress changeait avec le temps. Les tailles d’effet standards ont été calculées à l’aide du *d* de Cohen.

**Résultats:**

Les niveaux moyens de stress et de détresse psychologique étaient élevés au début de l’étude, et sont demeurés élevés aux trois mesures. Un niveau de stress semblablement élevé a été observé en moyenne pour les facteurs de stress spécifiques des étudiants. Alors que des différences significatives de la gravité moyenne aient été observées avec le temps pour certains facteurs de stress, les tailles d’effet standardisées étaient négligeables, ce qui suggère très peu de changement important et des niveaux constants de stress chronique au cours de l’année scolaire.

**Conclusions:**

Cet article est le premier qui examine les tendances du stress spécifique des étudiants à l’aide d’un échantillon national d’étudiants canadiens de niveau post-secondaire durant la première année de la pandémie de la COVID-19. Les modèles observés des facteurs de stress spécifiques des étudiants reflétaient des changements probablement indicateurs de la pandémie, notamment le stress le plus grave associé aux études, aux finances, et aux préoccupations quant à l’avenir. Les implications pour la future recherche sont discutées, en particulier, l’importance d’examiner les facteurs de stress reliés à la COVID-19 et leur effet sur la santé mentale des étudiants.

## Background

Post-secondary students face many stressors that place them at risk for mental health and academic outcomes that may have lifelong or life-threatening consequences, including poor academic performance, onset of mental illnesses, substance misuse, risk-taking behaviours and suicidality.^1–4^ Among first-year university students, high perceived stress has been identified as an important proximal risk factor for the development of symptoms of common mental illnesses (e.g., anxiety, depression) and poor academic outcomes (e.g., poor academic performance, difficulty adjusting to post-secondary setting).^2^ Importantly, stressors experienced by students throughout their post-secondary careers extend beyond those related to academics, spanning the learning environment, campus culture, interpersonal and personal/self-care factors, in addition to traditional academics.^5, 6^ In 2020, the global COVID-19 pandemic introduced a new layer of challenges for students. New stressors, ranging from an unexpected need to pivot to online/remote learning, interrupted academic activities including research and course work, isolation and loneliness stemming from campus closures, and economic uncertainty (e.g., loss of employment, potential loss of academic funding) were added to the growing list of stressors already experienced in the post-secondary setting.^7–9^ While research suggests that some degree of stress can occasionally promote improved performance, chronic, ongoing stress with little to no alleviation can result in the development of psychological distress without proper mitigation.^10^

Data collected from Canadian post-secondary institutions in 2019 through the National College Health Assessment (NCHA II) revealed large proportions of students reporting their past-year stress level to be “more than average” (45.6%) or “tremendous” (15.3%).^11^ Additionally, many students reported feeling hopeless (63.6%), overwhelmed (88.2%) and anxious (68.9%).^11^ Nearly a quarter of students reported having received a past-year diagnosis of anxiety (24%) or depression (20%), with 16% reporting a dual diagnosis.^11^ These levels of stress and psychological distress appear to have increased significantly between the 2013 and 2019 iterations of the NCHA II survey.^12^ Alongside the increasing prevalence of symptoms of common mental illnesses as well as self-reported diagnoses, an increase in the demand for student mental health services also has been documented, with demand outpacing available services.^13^ In response to increasing demands, many institutions have expanded on-campus treatment options,^14–16^ though few have attempted a whole campus approach to create a health-promoting university, as is described in the Okanagan Charter. Universities adopting Charter principles are encouraged to incorporate health (and mental health) into the university culture, processes and policies to promote an environment that enhances health and well-being.^17^ The addition of COVID-19-related stressors to the myriad of those already faced by students is concerning, particularly given that population-based data collected by Mental Health Research Canada (MHRC) suggests rates of depression and anxiety within the general Canadian population have tripled and quadrupled, respectively, since the onset of the pandemic,^18^ with young Canadians most likely to report deteriorating mental health.^19^

Existing upstream campus mental health resources could be greatly improved with better knowledge of student stressors, particularly in the wake of COVID-19. Indeed, assessing the ever-changing and diverse needs of students should be an important and ongoing process.^20^ Until recently, there has been no way to systematically catalogue the frequency and severity of these stressors. Existing instruments designed to evaluate student stress were either too narrowly conceived, seldom involved a wide range of students in the development process, or were too broad, including stress-related items that were not relevant to the post-secondary setting. Instruments did not capture a holistic picture of the stressors students currently face, and many presented poor or inappropriate psychometric properties.^21^

In order to fill this gap, the Post-Secondary Student Stressors Index (PSSI) was developed in collaboration with students. The tool evaluates 46 stressors pertinent to post-secondary life across 5 domains: academics, the learning environment, campus culture, interpersonal stressors and personal stressors.^22^ In a study examining proof-of-concept in a sample of over 500 students at one Ontario university, many of the most severe stressors were observed in the academic domain. Female students consistently rated all stressors as more severe than their male counterparts and also reported higher frequency ratings, indicating that they worried about them more often.^21^ Because these results were based on a moderately small sample from a single university, they may not be generalizable or useful for policy change.

In this paper, we report results from a larger study that used the PSSI to collect longitudinal data on self-reported stressors across 15 Canadian universities over the course of the 2020–2021 academic year. In addition to describing the most severe and frequently occurring stressors, the intended purpose of this paper was to examine whether the distribution of severe stressors changed significantly and meaningfully over the course of the academic year. Although evaluating student stress during the COVID-19 pandemic was not an original goal, this study was coincidentally conducted during the first complete academic year that took place during the pandemic. As a result, the findings described here are interpreted in context of the pandemic and consider how this global event may have influenced students’ experiences of stress within the post-secondary setting during this time. Results will provide a broader view of the nature of stressors experienced by Canadian university students and provide a stronger platform for targeted upstream campus mental health services in the wake of the ongoing COVID-19 pandemic.

## Methods

### Study Design and Procedure

A repeated measures cross-sectional study focusing on university student stress and mental health outcomes was completed during the 2020–2021 academic year, collecting data at three time points through the use of online surveys. T1 took place in October 2020, T2 in January 2021 and T3 in late March or early April 2021. Participants were students attending 15 post-secondary institutions across Canada, representing 9 provinces (all but Quebec) and the Northwest Territories. Survey questions included demographics; sources of post-secondary student stress and degree of stress experienced; and mental health-related measures, including general perceived stress, resilience, psychological distress and history of mental illness diagnoses. Surveys remained open for approximately 3 weeks, with one reminder sent (or request for participants refreshed). Participants reviewed a letter of information prior to completing each survey, providing implied consent through survey submission. The sampling method and sample size used was at the discretion of each participating institution (see Supplemental material A). Although allowing different sampling strategies to be used increases the risk of bias, a sensitivity analysis of the sample demographics and stressor assessments did not reveal any concerns. Additionally, the researchers found that allowing partner institutions with more restrictive student data sharing policies with the flexibility to administer the survey using their preferred method resulted in more overall university partners participating in this cross-Canada study. Additional details on the study procedures have been published elsewhere.^23^ Ethics approval was obtained from Queen's University's Health Sciences and Affiliated Teaching Hospitals Research Ethics Board (#6029173) as the lead university, as well as the ethics boards at each participating university.

### Measures

The survey collected the following demographic information: age, gender, level of study (undergraduate, graduate or professional programme), international student status (yes/no), first-generation student status (yes/no) and region of institution (Northern Canada, Atlantic Canada, Western Canada or Central Canada). All demographic variables with the exception of age were categorical in nature. Due to small cell counts, the 14 participants from the Northern region of Canada were excluded from analysis.

Stress and psychological distress were evaluated by the Perceived Stress Scale (PSS-10)^24^ and Kessler Psychological Distress Scale (K10),^25^ respectively. The PSS-10 is designed to measure overall perceived stress, with response options ranging from 0 (never) to 4 (very often). The K10 is designed to measure non-specific psychological distress, with response options ranging from 1 (none of the time) to 5 (all of the time). On both scales, responses are summed to derive composite scores, with higher scores indicative of greater perceived stress and psychological distress, respectively. Both the PSS-10 and K10 have demonstrated strong psychometric properties when used among samples of post-secondary populations.^26–28^

Student-specific stressors were assessed by the PSSI, a 46-item index listing stressors across five domains: academics, the learning environment, campus culture, the interpersonal and the personal. Respondents are asked to rate each stressor by both severity and frequency of stress on an adjectival scale from 1 (“not stressful” and “rarely”) to 4 (“extremely stressful” and “almost always”). An additional option to indicate N/A was provided in the event that a stressor was not applicable or did not occur (scored as zero). Preliminary evaluation has demonstrated strong psychometric properties for the tool, including test–retest reliability and strong relationships with like constructs among a sample of students at an Ontario university.^5^

### Analysis

Descriptive statistics (frequencies and measures of central tendency and dispersion, where appropriate) were calculated for all variables. Means for severity and frequency were calculated for all stressor variables on the PSSI, excluding responses of ‘N/A’. Kruskal–Wallis ranked tests were performed to assess whether the mean severity for each stressor changed across the three time points. Multiple pairwise comparison analyses (Wilcoxon rank-sum tests) were then conducted to determine at which time points mean severity differed significantly. We elected not to use a *P*-value correction for multiple comparisons per Perneger's recommendation.^29^ Standard effect sizes were calculated for each time point comparison using Cohen's *d*.

### Missing Data

Participants who indicated that a stressor was not applicable to them or that it was not experienced were excluded from analyses; these responses would be invalid to include in calculations determining the severity of stress stemming from a stressor that was not experienced by the respondent. A complete case analysis approach was taken to manage missing data, where any participant who had complete information for the variables relevant to each statistical test was included in the analysis. A Supplementary table reporting the degree of missing data for each test as well as the number of participants who indicated they had *not* experienced a stressor can be found in Supplemental material B.

## Results

### Sample

Table 1 presents the demographics for the samples at each data collection time point. There were similar trends across time points, with each group being primarily female and under the age of 25 years. At the first time point, the largest proportion of respondents were 20 years or younger (42.7%), dropping off proportionally at T2 (29.8%) and T3 (25.9%). The average age ranged from 23.4 (*median* = 21, *SD*  =  6.8) to 24.9 (*median* = 22, *SD*  =  7.0). Most participants were full-time, undergraduate students with an estimated GPA around A. The majority of students who participated attended university in the Atlantic and Western regions of Canada, reported a marital status of “single”, and lived off-campus with family or roommates.

**Table 1.** Sociodemographic Characteristics of Participants Across Time Points.

	T1 (N = 4,954) n (%)	T2 (N = 4,576) n (%)	T3 (N = 3,083) n (%)
**Region of Institution**			
Northern Canada^a^	14 (0.3)	11 (0.3)	11 (0.4)
Atlantic Canada	2,014 (40.7)	1,235 (28.4)	1,115 (37.3)
Western Canada	1,952 (39.4)	2,235 (51.4)	1,254 (42.0)
Central Canada	972 (19.6)	867 (19.9)	606 (20.3)
**Sex**			
Female	3,584 (72.3)	3,292 (71.9)	2,164 (70.2)
Male	1,240 (25.0)	1,145 (25.0)	813 (26.4)
Non-binary	83 (1.7)	90 (2.0)	65 (2.1)
Prefer not to answer	47 (0.9)	49 (1.1)	40 (1.3)
**Age**			
≤ 20 years	2,116 (42.7)	1,360 (29.8)	798 (25.9)
21–24 years	1,546 (31.2)	1,735 (38.0)	1,210 (39.3)
25–29 years	635 (12.8)	776 (17.0)	558 (18.1)
≥ 30 years	654 (13.2)	698 (15.3)	511 (16.6)
* Mean (SD)*	*23.41* (*6.90)*	*24.51* (*6.85)*	*24.89* (*7.04)*
**Level of Study**			
Undergraduate	3,774 (76.2)	3,430 (75.0)	2,259 (73.3)
Graduate	889 (17.9)	871 (19.0)	607 (19.7)
Professional Program	166 (3.4)	154 (3.4)	146 (4.7)
Other	107 (2.2)	105 (2.3)	55 (1.8)
Prefer not to answer	18 (0.4)	16 (0.3)	16 (0.5)
**Relationship Status**			
Single	4,255 (85.9)	3,909 (85.4)	2,619 (84.9)
Married/common-law	591 (11.9)	584 (12.8)	404 (13.1)
Separated, divorced or widowed	52 (1.0)	30 (0.7)	31 (1.0)
Prefer not to answer	56 (1.1)	53 (1.2)	29 (0.9)
**Residence**			
On campus residence	423 (8.5)	328 (7.2)	218 (7.1)
Off campus with roommates	1,360 (27.5)	1,167 (25.5)	821 (26.6)
Off campus alone	499 (10.1)	455 (9.9)	315 (10.2)
Off campus with family	2,588 (52.2)	2,552 (55.8)	1,672 (54.2)
Prefer not to answer	84 (1.7)	71 (1.6)	57 (1.8)
**Student Status**			
Full-time	4,548 (91.8)	4,126 (90.2)	2,747 (89.1)
Part-time	369 (7.4)	404 (8.8)	295 (9.6)
Other	26 (0.5)	38 (0.8)	28 (0.9)
Prefer not to answer	11 (0.2)	8 (0.2)	13 (0.4)
**First-Generation Student**			
Yes	1,330 (26.8)	1,186 (25.9)	757 (24.6)
No	3,595 (72.6)	3,365 (73.5)	2,313 (75.0)
Prefer not to answer	29 (0.6)	25 (0.5)	13 (0.4)
**International Student**			
Yes	585 (11.8)	628 (13.7)	377 (12.2)
No	4,358 (88.0)	3,938 (86.1)	2,702 (87.6)
Prefer not to answer	11 (0.2)	10 (0.2)	4 (0.1)
**Estimated GPA Range**			
A	2,996 (60.6)	2,713 (59.3)	1,895 (61.5)
B	1,386 (28.0)	1,333 (29.1)	896 (29.1)
C	299 (6.0)	304 (6.6)	179 (5.8)
D/F	14 (0.3)	28 (0.6)	19 (0.6)
Prefer not to answer	253 (5.1)	197 (4.3)	94 (3.0)

***Notes*.** (1) Total sample reported here is unmatched.

(2) ^a^Northern Canadian participants were removed from subsequent analyses due to small cell counts.

### Univariate Analysis of Stress and Distress

The average K10 score among this sample was 28.66 (*SD* = 9.54) at T1, with this level of distress maintained over the course of the academic year (T2 $\bar{X}$ = 28.46, *SD* = 9.44; T3 $\bar{X}$ = 29.22, *SD* = 9.66). At T1, the average PSS-10 score was 23.74 (*SD* = 3.93), maintained across time points (T2 $\bar{X}$ = 23.31, *SD* = 3.96; T3 $\bar{X}$ = 23.49, *SD* = 3.97). A similarly high level of stress was observed on average for PSSI stressors over time.

The mean severity and frequency of each stressor on the PSSI across time points is reported in Supplemental material C. Across time points, stressors within the academic domain were consistently rated as the most severe on average. Within the learning environment, stressors related to lack of clarity in instruction and expectations for students were rated as most severe on average, while “pressure to succeed” and “worrying I’m not working hard enough” (campus culture domain) and “meeting my own expectations” (interpersonal domain) were the most frequently occurring stressors on average, in addition to being some of the most severe. Within the personal domain, stressors associated with finances (i.e., debt, worrying about getting a job after graduation, having to take loans) emerged as some of the most severe, in addition to those related to work/life balance (i.e., feeling guilty about taking time for hobbies/interests, balancing work with academics). Overall, mean severity ratings ranged from 1.86 (“meeting with my professor”) to 3.44 (“writing multiple exams around the same time”), with few falling below 2.

### Changes in Stress Over Time

Statistically significant mean differences in the severity of stress across time points were observed for several stressors across domains (Table 2). The majority of significant differences were observed within the academic domain, including having exams worth ≥50% of course grade (*p*  =  0.01), heavily weighted assignments (*p*  =  0.04), multiple assignments due around the same time (*p*  =  0.01), managing academic workload (*p* < 0.001), receiving a bad grade (*p*< 0.001) and maintaining GPA (*p*  =  0.004). Within the learning environment domain, only the mean severity for one stressor varied significantly over time (meeting with my professor, *p* < .001), while three mean differences were statistically significant within the campus culture domain (adjusting to the post-secondary lifestyle (*p* < 0.001); feeling like I’m not working hard enough (*p* =  0.001); and pressure to succeed, *p* < 0.001). Within the interpersonal domain, only mean severity for balancing social life with academics (*p* < 0.001) and meeting my own expectations (*p* < 0.001) varied significantly over time, while three mean differences were statistically significant within the personal domain (having to prepare meals for myself, *p*  =  0.02; balancing work with academics, *p* < 0.001; and feeling guilty about taking time for hobbies/interests, *p* < 0.001).

**Table 2.** Mean Severity Differences Across Time Points.

	Mean Severity			Kruskal–Wallis (Any difference)		Wilcoxon Rank-sum Test (Difference between groups)		
Stressor				**χ^2^_df_ _=_ _2_**		*P*		
*Academic Domain*	T1	T2	T3			T1/T2	T2/T3	T1/T3
Preparing for exams	2.75	2.73	2.73	2.76	0.25	0.12	0.90	0.22
Writing exams	2.78	2.77	2.80	2.35	0.31	0.91	0.16	0.18
Writing multiple exams around the same time	3.44	3.41	3.42	3.04	0.22	0.09	0.58	0.34
Exams worth ≥50% of course grade	3.35	3.29	3.31	9.71	**0**.**01**	**0**.**002**	0.45	0.05
Heavily weighted assignments	2.66	2.62	2.66	6.41	**0**.**04**	**0**.**03**	0.75	**0**.**03**
Having multiple assignments due around the same time	2.97	2.97	3.03	9.34	**0**.**01**	0.96	**0**.**01**	**0**.**01**
Managing my academic workload	2.63	2.54	2.61	26.20	**<0**.**001**	**<0**.**001**	**<0**.**001**	0.44
Receiving a bad grade	3.16	3.13	3.08	17.65	**<0**.**001**	0.08	**0**.**01**	**<0**.**001**
Maintaining my GPA	2.81	2.75	2.75	11.31	**0**.**004**	**0**.**003**	0.99	**0**.**01**
Working on my thesis	2.73	2.75	2.78	2.11	0.35	0.48	0.41	0.15
Performing well at my placement (i.e., practicum)	2.52	2.54	2.57	1.81	0.41	0.51	0.45	0.18
***Learning Environment Domain***								
Poor communication from professor	2.84	2.83	2.81	2.54	0.28	0.64	0.25	0.12
Lack of clarity from professor	2.93	2.91	2.91	2.63	0.27	0.16	0.95	0.19
Lack of guidance from professor	2.80	2.79	2.79	1.50	0.47	0.24	0.83	0.41
Meeting with my professor	1.86	1.92	1.95	15.06	**<0**.**001**	**0**.**01**	0.22	**<0**.**001**
Meeting my thesis/placement supervisor's expectations	2.44	2.44	2.45	0.08	0.96	1.00	0.81	0.80
Lack of mentoring from my thesis/placement supervisor	2.49	2.52	2.52	0.96	0.62	0.34	0.84	0.53
***Campus Culture Domain***								
Adjusting to the post-secondary lifestyle	2.07	2.03	1.98	18.67	**<0**.**001**	0.19	**0**.**002**	**<0**.**001**
Adjusting to my programme	2.06	2.02	2.02	5.12	0.08	0.06	0.76	0.05
Academic competition among my peers	2.02	2.03	2.02	1.79	0.41	0.19	0.42	0.72
Feeling like I’m not working hard enough	2.87	2.81	2.80	13.08	**0**.**001**	**0**.**002**	0.87	**0**.**003**
Feeling like my peers are smarter than me	2.54	2.52	2.50	34.64	0.18	0.22	0.49	0.08
Pressure to succeed	3.01	2.95	2.94	16.11	**<0**.**001**	**<0**.**001**	0.53	**<0**.**001**
Discrimination on campus	1.97	2.00	2.03	2.36	0.31	0.47	0.38	0.13
Sexual harassment on campus	2.18	2.22	2.27	5.44	0.07	0.16	0.33	**0**.**02**
***Interpersonal Domain***								
Making new friends	2.05	2.06	2.04	0.59	0.75	0.64	0.45	0.73
Maintaining friendships	1.95	1.96	1.99	3.50	0.18	0.70	0.15	0.07
Networking with the “right” people	2.15	2.19	2.19	4.03	0.13	0.06	0.86	0.14
Feeling pressured to socialize	2.10	2.11	2.10	0.93	0.63	0.40	0.43	0.97
Balancing a social life with academics	2.52	2.41	2.46	32.99	**<0**.**001**	**<0**.**001**	**0**.**04**	**0**.**003**
Comparing myself to others	2.62	2.61	2.60	1.24	0.54	0.50	0.63	0.28
Comparing myself to others on social media	2.21	2.21	2.23	1.19	0.55	0.88	0.36	0.30
Meeting others peoples’ expectations of me	2.54	2.51	2.55	4.42	0.11	0.09	0.06	0.71
Meeting my own expectations	3.07	3.00	3.03	16.27	**<0**.**001**	**<0**.**001**	0.21	**0**.**02**
***Personal Domain***								
Making sure that I get enough sleep	2.31	2.27	2.28	3.79	0.15	0.05	0.37	0.39
Making sure that I get enough exercise	2.35	2.32	2.37	4.57	0.10	0.19	0.05	0.34
Making sure that I eat healthy	2.27	2.26	2.29	2.51	0.28	0.32	0.12	0.50
Having to prepare meals for myself	1.95	1.94	2.01	7.56	**0**.**02**	0.98	**0**.**01**	**0**.**01**
Balancing working at my job with academics	2.73	2.63	2.67	19.16	**<0**.**001**	**<0**.**001**	0.06	0.05
Balancing my extracurriculars with academics	2.22	2.17	2.21	6.23	0.05	**0**.**02**	0.08	0.77
Feeling guilty about taking time for my hobbies/interests	2.66	2.51	2.63	53.68	**<0**.**001**	**<0**.**001**	**<0**.**001**	0.22
Having to take student loans	2.79	2.73	2.75	4.37	0.11	**0**.**04**	0.60	0.22
Worrying about paying off debt	2.85	2.80	2.82	2.14	0.34	0.14	0.52	0.54
Worrying about getting a job after graduating	2.83	2.88	2.87	4.64	0.10	**0**.**04**	0.64	0.16
Worrying about getting into a new programme after graduating	2.74	2.71	2.72	2.31	0.31	0.13	0.58	0.45
Worrying about missing major “life events” (i.e., buying a house, marriage, children)	2.71	2.72	2.74	1.58	0.45	0.61	0.43	0.21

***Notes*.** (1) Bolded *P*-values are statistically significant at a 95% confidence level.

(2) Wilcoxon rank-sum tests performed without *P*-value adjustments for multiple comparisons.

Despite the statistical differences observed, standardized effect sizes revealed negligible results with Cohen's *d* values ranging from 0.01 to 0.15 (Table 3). These values fall short of the recommended cut-off for even a small effect size.^30^ That said, the largest effect sizes were observed between the first and final time points (i.e., the start and end of the academic year) or the first and second time points (i.e., the start and end of the first semester). In particular, the following stressors demonstrate effect sizes over 0.10 between T1 and T3: exams worth ≥50% of course grade (*d* = 0.14), receiving a bad grade (*d* = 0.12) and adjusting to the post-secondary lifestyle (*d* = 0.15). Between T1 and T2, two stressors demonstrated effect sizes over 0.10: balancing social life with academics (*d* = 0.12) and feeling guilty about taking time for hobbies/interested (*d* = 0.12). Notably, these stressors were also among the highest rated by severity.

**Table 3.** Standardized Effect Sizes for Stressors Where a Statistically Significant Difference was Observed Over Time.

	Mean Severity			Cohen's *d*		
Stressor				*d*		
	T1	T2	T3	T1/T2	T2/T3	T1/T3
***Academic Domain***						
Exams worth ≥50% of course grade	3.35	3.29	3.31	0.07	0.06	**0**.**13**
Having multiple assignments due around the same time	2.97	2.97	3.03	0.03	0.04	0.01
Managing my academic workload	2.63	2.54	2.61	**0**.**10**	0.07	0.02
Receiving a bad grade	3.16	3.13	3.08	0.08	0.03	**0**.**12**
Maintaining my GPA	2.81	2.75	2.75	0.07	0.01	0.08
***Learning Environment Domain***						
Meeting with my professor	1.86	1.92	1.95	0.04	0.01	0.04
***Campus Culture Domain***						
Adjusting to the post-secondary lifestyle	2.07	2.03	1.98	0.06	0.09	**0**.**15**
Feeling like I’m not working hard enough	2.87	2.81	2.80	0.06	0.01	0.05
Pressure to succeed	3.01	2.95	2.94	0.05	0.02	0.07
***Interpersonal Domain***						
Balancing social life with academics	2.52	2.41	2.46	**0**.**12**	0.03	0.09
Meeting my own expectations	3.07	3.00	3.03	0.08	0.03	0.05
***Personal Domain***						
Having to prepare meals for myself	1.95	1.94	2.01	0.02	0.05	0.07
Balancing working at my job with academics	2.73	2.63	2.67	0.04	0.01	0.04
Feeling guilty about taking time for my hobbies/interests	2.66	2.51	2.63	**0**.**12**	0.11	0.01

***Note*.** (1) Cohen's *d* is calculated at 95% level of confidence.

## Discussion

To our knowledge, this is the first paper to examine longitudinal trends in student-specific stressors using a nationwide sample of Canadian post-secondary students during the first complete academic year of the COVID-19 pandemic. While we did observe statistically significant differences in mean severity for several stressors in each domain, standardized effect sizes were negligible, suggesting that the mean severity of stress did not change meaningfully for students over the course of the semester. The observed statistical significance is likely attributable to sample size, rather than being indicative of meaningful change.

Patterns observed in student-specific stressors reflected changes that may be indicative of the COVID-19 pandemic. Self-care items in the personal domain, including “having to cook meals for myself”, “getting enough sleep”, “eating nutritious foods” were rated lower than others on average, perhaps due to the fact that the majority of students in this sample reported living at home with family during this academic year, likely due to campus closures. Similarly, stressors related to instruction, including “lack of clarity” and “poor communication” from the instructor were rated highly, which may reflect the added stress of unexpected, mandatory online learning experienced by students.^9^ Within the campus culture domain, “feeling like I’m not working hard enough” was the most highly rated stressor, which may reflect students having difficulty adjusting to working from home, outside of the more collegiate, post-secondary setting. Within the interpersonal domain, “meeting my own expectations” and “comparing myself to others” were rated quite highly, while ratings for stressors related to managing friendships and pressure to socialize were much lower than what was observed during the pilot study of the instrument.^21^ Again, these findings are consistent with being removed from the more social setting of university life. Notably, “balancing a social life with academics” was rated higher than the other social stressors, likely reflecting the challenges associated with repeated requirements to self-isolate and quarantine. Finally, both financial concerns (i.e., “paying off debt”, “having to take student loans”) and concerns for the future (i.e., “getting into a new program”, “getting a job after graduating”) were rated highly, perhaps owing to the fact that many students were unable to find employment during this time.^9^ Overall, stressors with the highest rated severity were related to academic and financial concerns, reflecting two areas of massive change for students during the pandemic. Future research should investigate stressors experienced by students specific to COVID-19 and their impact on overall psychological distress to inform institutions’ efforts in supporting students’ mental health and well-being moving forward throughout the ongoing pandemic and its eventual aftermath.

It is important to note that this sample of students demonstrated a high level of overall stress and psychological distress. Average scores on the PSS-10 and K10 were high at the first data collection time point and remained high over the course of the semester, echoing the mean severity of stressors on the PSSI. In fact, the averages observed were considerably higher than K10 and PSS-10 scores observed in previous studies among post-secondary students.^31–35^ Overall, the severity of stress and distress experienced by this nationwide sample of post-secondary students was high at the start of the academic year and remained so over the course of the year. This consistent level of stress was surely influenced by the COVID-19 pandemic, consistent with what was observed at the population level during the same time period. For example, in October 2020, Canadians reported high levels of psychological distress, with one in four reporting a diagnosis of anxiety or depression and more than half of poll participants indicating moderate-to-severe psychological distress.^36^ Canadians reported their highest levels of anxiety and depression around December 2020, surpassing the highest levels seen up until that point, which were recorded at the beginning of the pandemic.^37^ Neurobiologically, chronic stress significantly increases the risk of developing a mental illness,^38, 39^ and post-secondary aged individuals (18–25 years) are particularly at risk due to their stage of brain development. Notably, the highest levels of anxiety and depression reported in June 2021 were observed among younger Canadians, with 20% of respondents aged 18–34 years reporting thoughts of suicide within the past year.^40^ Given that suicide remains the leading cause of death among young Canadians,^41^ these findings are particularly concerning.

### Limitations

The results of this study should be interpreted in light of a few notable limitations. Although data for this study was collected at multiple points over the course of one academic year, the results presented here were derived from cross-sectional (i.e., unmatched) data rather than linked longitudinal data. Second, the data is subject to self-report bias, creating the potential for over- or underestimates of true levels of stress and distress. Volunteer bias is also likely present in our sample with the vast majority of respondents being female and reporting a high GPA in the A range. It is likely that higher-achieving students were more willing to volunteer for research compared to their lower-achieving counterparts. It is also feasible that students experiencing the most severe levels of stress and psychological distress would be the least likely to participate, meaning that the results presented here may be underestimates of the true effect. Finally, while we can make inferences about the potential contributions of the COVID-19 pandemic with respect to patterns we observed in student stressors, the impact of the pandemic is not directly measured. In this study, we did not assess patterns in stressors across the different regions of Canada with respect to pandemic response, restrictions and the timing of virus waves, which will be an important area for future research to explore.

## Conclusion

The chronic stress brought on by the COVID-19 pandemic and ensuing mental health issues continues to be problematic. While the most recent poll conducted by MHRC showed that the majority of Canadians are now fully vaccinated (and many boosted) against the virus, levels of self-reported anxiety and depression have decreased only slightly since December 2021, with about one-fifth of Canadians continuing to report high levels of distress^18^ Our study showed that students’ levels of stress and psychological distress remained high over the entirety of the 2020–2021 academic year. No meaningful change in the mean severity of stress experienced by student-specific stressors on the PSSI was evident over time. Moving forward, it will be important to explore the continued impact of COVID-19 on student stress to inform post-secondary institutions’ efforts to bolster upstream mental health resources to support students’ emotional well-being, including targeting key areas such as academics and financial literacy.

## Supplemental Material

sj-docx-1-cpa-10.1177_07067437221111365 - Supplemental material for Trends in Post-Secondary Student Stress: A Pan-Canadian Study Click here for additional data file. Supplemental material, sj-docx-1-cpa-10.1177_07067437221111365 for Trends in Post-Secondary Student Stress: A Pan-Canadian Study by Brooke Linden, Heather Stuart and Amy Ecclestone in The Canadian Journal of Psychiatry
