# Supplementary material for: Trends in Post-Secondary Student Stress: A Pan-Canadian Study
Source: Can J Psychiatry. 2022 Jul 6;68(7):521–30. doi: 10.1177/07067437221111365 (PMC10408557; doi:10.1177/07067437221111365)
Supplement: sj-docx-1-cpa-10.1177_07067437221111365 - Supplemental material for Trends in Post-Secondary Student Stress: A Pan-Canadian Study [file sj-docx-1-cpa-10.1177_07067437221111365.docx]

**Supplement A**

*Table A-1. Number of Responses at Participating Institutions over Time and Sampling Methods*

| **Institution** | **T1**  **n** | **T2**  **n (%)** | **T3**  **n (%)** | **Sampling Method** |
| --- | --- | --- | --- | --- |
| Aurora College (1) | 14 | 11 | 11 | Listserv |
| UBC Okanagan (9) | 152 | 109 | 55 | Listserv |
| University of Alberta (8) | 0 | 227 | 97 | E-mail Newsletter |
| University of Calgary (10) | 150 | 87 | 57 | E-mail Newsletter |
| University of Saskatchewan (16) | 64 | 51 | 22 | PAWS |
| University of Regina (15) | 346 | 466 | 235 | Listserv |
| University of Manitoba (11) | 1240 | 1522 | 885 | Listserv |
| Lakehead University (3) | 271 | 230 | 166 | Listserv |
| Western University (17) | 439 | 363 | 279 | Listserv |
| Queen’s University (6) | 262 | 274 | 161 | Student Sample |
| University of New Brunswick (12) | 1147 | 529 | 435 | Listserv |
| University of PEI (14) | 55 | 11 | 2 | Social Media, Campus Notices |
| St. Francis Xavier University (7) | 26 | 0 | 0 | SONA |
| Dalhousie University (2) | 2 | 1 | 0 | SONA |
| Memorial University of NFLD (5) | 784 | 694 | 678 | Listserv |

**Supplement B**

*Table B-1. Quantity of Missing Data and Responses of N/A for PSSI Stressors Variables over Time*

|  | **T1** | | **T2** | | **T3** | |
| --- | --- | --- | --- | --- | --- | --- |
| **Stressor** | Missing  *n* | N/A  *n (%)* | Missing  *n* | N/A  *n (%)* | Missing  *n* | N/A  *n (%)* |
| ***Academic Domain*** |  |  |  |  |  |  |
| Preparing for exams | 0 | 323 (6.5) | 0 | 332 (7.3) | 0 | 267 (8.7) |
| Writing exams | 2 | 360 (7.3) | 1 | 339 (7.4) | 1 | 274 (8.9) |
| Writing multiple exams around the same time | 1 | 835 (16.9) | 3 | 987 (21.6) | 3 | 708 (23.0) |
| Exams worth more than 50% of course grade | 0 | 740 (15.0) | 1 | 760 (16.7) | 1 | 604 (19.7) |
| Heavily weighted assignments | 0 | 138 (2.8) | 0 | 172 (3.8) | 0 | 119 (3.9) |
| Multiple assignments due around the same time | 2 | 121 (2.5) | 2 | 169 (3.7) | 2 | 126 (4.1) |
| Managing my academic workload | 2 | 23 (0.5) | 2 | 25 (0.5) | 2 | 22 (0.7) |
| Receiving a bad grade | 4 | 228 (4.6) | 7 | 296 (6.5) | 7 | 195 (6.4) |
| Maintaining my GPA | 13 | 135 (2.7) | 10 | 152 (3.3) | 10 | 110 (3.6) |
| Working on my thesis | 7 | 3194 (64.7) | 6 | 2977 (65.3) | 6 | 2108 (68.7) |
| Performing well at my placement (i.e., practicum) | 2 | 2827 (57.2) | 2 | 2620 (57.4) | 2 | 1801 (58.6) |
| ***Learning Environment Domain*** |  |  |  |  |  |  |
| Poor communication from professor | 0 | 260 (5.3) | 1 | 292 (6.4) | 0 | 189 (6.2) |
| Lack of clarity from professor | 0 | 196 (4.0) | 0 | 216 (4.7) | 0 | 149 (4.9) |
| Lack of guidance from professor | 1 | 246 (5.0) | 1 | 265 (5.8) | 0 | 182 (5.9) |
| Meeting with my professor | 7 | 636 (12.9) | 8 | 587 (12.9) | 5 | 437 (14.2) |
| Meeting my supervisor’s expectations | 2 | 2995 (60.7) | 2 | 2736 (60.0) | 0 | 1949 (63.4) |
| Lack of mentoring from my supervisor | 1 | 3236 (65.5) | 1 | 3006 (65.9) | 2 | 2083 (67.9) |
| ***Campus Culture Domain*** |  |  |  |  |  |  |
| Adjusting to the post-secondary lifestyle | 0 | 270 (5.5) | 0 | 330 (7.2) | 1 | 274 (8.9) |
| Adjusting to my program | 2 | 156 (3.2) | 1 | 203 (4.4) | 0 | 170 (5.5) |
| Academic competition among my peers | 3 | 374 (7.6) | 2 | 377 (8.3) | 0 | 271 (8.8) |
| Feeling like I’m not working hard enough | 1 | 148 (3.0) | 1 | 148 (3.2) | 0 | 81 (2.6) |
| Feeling like my peers are smarter than me | 0 | 244 (4.9) | 0 | 216 (4.7) | 1 | 170 (5.5) |
| Pressure to succeed | 2 | 69 (1.4) | 0 | 58 (1.3) | 8 | 43 (1.4) |
| Discrimination on campus | 2 | 2919 (59.1) | 2 | 2749 (60.2) | 1 | 1820 (59.3) |
| Sexual harassment on campus | 0 | 3193 (64.6) | 2 | 3022 (66.2) | 1 | 1956 (63.7) |
| ***Interpersonal Domain*** |  |  |  |  |  |  |
| Making new friends | 0 | 339 (6.9) | 0 | 368 (8.1) | 0 | 308 (10.0) |
| Maintaining friendships | 0 | 200 (4.0) | 0 | 187 (4.1) | 0 | 142 (4.6) |
| Networking with the “right” people | 2 | 451 (9.1) | 2 | 439 (9.6) | 1 | 329 (10.7) |
| Feeling pressured to socialize | 2 | 493 (10.0) | 0 | 491 (10.8) | 4 | 366 (11.9) |
| Balancing a social life with academics | 0 | 140 (2.8) | 2 | 151 (3.3) | 1 | 118 (3.8) |
| Comparing myself to others | 0 | 249 (5.0) | 2 | 218 (4.8) | 2 | 172 (5.6) |
| Comparing myself to others on social media | 3 | 756 (15.3) | 1 | 658 (14.4) | 0 | 494 (16.1) |
| Meeting others peoples’ expectations of me | 2 | 256 (5.2) | 0 | 241 (5.3) | 0 | 163 (5.3) |
| Meeting my own expectations | 0 | 35 (0.7) | 2 | 41 (0.9) | 0 | 22 (0.7) |
| ***Personal Domain*** |  |  |  |  |  |  |
| Making sure that I get enough sleep | 1 | 56 (1.1) | 1 | 56 (1.2) | 0 | 35 (1.1) |
| Making sure that I get enough exercise | 0 | 147 (3.0) | 0 | 114 (2.5) | 0 | 77 (2.5) |
| Making sure that I eat healthy | 5 | 77 (1.6) | 0 | 76 (1.7) | 1 | 39 (1.3) |
| Having to prepare meals for myself | 8 | 357 (7.2) | 5 | 273 (6.0) | 1 | 197 (6.4) |
| Balancing working at my job with academics | 2 | 1621 (32.8) | 2 | 1485 (32.5) | 4 | 1024 (33.4) |
| Balancing my extracurriculars with academics | 2 | 1209 (24.5) | 2 | 1156 (25.3) | 0 | 850 (27.7) |
| Feeling guilty about taking time for my hobbies/interests | 1 | 273 (5.5) | 1 | 249 (5.5) | 2 | 154 (5.0) |
| Having to take student loans | 6 | 2433 (49.3) | 3 | 2340 (51.3) | 4 | 1633 (53.2) |
| Worrying about paying off debt | 5 | 1965 (39.8) | 4 | 1838 (40.3) | 4 | 1340 (43.7) |
| Worrying about getting a job after graduating | 5 | 418 (8.5) | 3 | 349 (7.7) | 4 | 270 (8.8) |
| Worrying about getting into a new program after graduating | 4 | 1414 (28.6) | 0 | 1374 (30.1) | 2 | 987 (32.1) |
| Worrying about missing major “life events”  (i.e., buying a house, marriage, children) | 4 | 449 (9.1) | 3 | 417 (9.1) | 0 | 316 (10.3) |

**Notes.** (1) “Missing” refers to non-responses; (2) “N/A” refers to responses of “Not applicable or this did not happen to me” for a stressor on the PSSI (these responses were coded as ‘missing’ for analyses); (3) Northern Canada observations removed.

**Supplement C**

*Table C-1. Stressors by Mean Severity and Mean Frequency across Time Points*

|  | **T1** | | **T2** | | **T3** | |
| --- | --- | --- | --- | --- | --- | --- |
| **Stressor** | **Sev** | **Freq** | **Sev** | **Freq** | **Sev** | **Freq** |
| ***Academic Domain*** |  |  |  |  |  |  |
| Preparing for exams | 2.57 | 2.55 | 2.53 | 2.52 | 2.53 | 2.52 |
| Writing exams | 2.78 | 2.53 | 2.77 | 2.50 | 2.77 | 2.50 |
| Writing multiple exams around the same time | 3.44 | 2.41 | 3.41 | 2.37 | 3.41 | 2.37 |
| Exams worth more than 50% of course grade | 3.35 | 2.54 | 3.29 | 2.51 | 3.29 | 2.51 |
| Heavily weighted assignments | 2.66 | 2.52 | 2.62 | 2.47 | 2.62 | 2.47 |
| Having multiple assignments due around the same time | 2.97 | 2.88 | 2.97 | 2.79 | 2.97 | 2.79 |
| Managing my academic workload | 2.63 | 2.95 | 2.54 | 2.85 | 2.54 | 2.85 |
| Receiving a bad grade | 3.16 | 2.49 | 3.13 | 2.42 | 3.13 | 2.42 |
| Maintaining my GPA | 2.81 | 2.77 | 2.75 | 2.72 | 2.75 | 2.72 |
| Working on my thesis | 2.73 | 2.61 | 2.75 | 2.65 | 2.75 | 2.65 |
| Performing well at my placement (i.e., practicum) | 2.52 | 2.46 | 2.54 | 2.49 | 2.54 | 2.49 |
| ***Learning Environment Domain*** |  |  |  |  |  |  |
| Poor communication from professor | 2.84 | 2.23 | 2.83 | 2.18 | 2.83 | 2.18 |
| Lack of clarity from professor | 2.93 | 2.31 | 2.91 | 2.24 | 2.91 | 2.24 |
| Lack of guidance from professor | 2.80 | 2.24 | 2.79 | 2.16 | 2.79 | 2.16 |
| Meeting with my professor | 1.86 | 1.69 | 1.92 | 1.72 | 1.92 | 1.72 |
| Meeting my thesis/placement supervisor’s expectations | 2.44 | 2.26 | 2.44 | 2.26 | 2.44 | 2.26 |
| Lack of mentoring from my thesis/placement supervisor | 2.49 | 2.09 | 2.52 | 2.09 | 2.52 | 2.09 |
| ***Campus Culture Domain*** |  |  |  |  |  |  |
| Adjusting to the post-secondary lifestyle | 2.07 | 1.98 | 2.03 | 1.97 | 2.03 | 1.97 |
| Adjusting to my program | 2.06 | 2.00 | 2.02 | 1.97 | 2.02 | 1.97 |
| Academic competition among my peers | 2.02 | 2.04 | 2.03 | 2.05 | 2.03 | 2.05 |
| Feeling like I’m not working hard enough | 2.87 | 2.89 | 2.81 | 2.82 | 2.81 | 2.82 |
| Feeling like my peers are smarter than me | 2.54 | 2.56 | 2.52 | 2.53 | 2.52 | 2.53 |
| Pressure to succeed | 3.01 | 2.99 | 2.95 | 2.92 | 2.95 | 2.92 |
| Discrimination on campus | 1.97 | 1.68 | 2.00 | 1.69 | 2.00 | 1.69 |
| Sexual harassment on campus | 2.18 | 1.66 | 2.22 | 1.69 | 2.22 | 1.69 |
| ***Interpersonal Domain*** |  |  |  |  |  |  |
| Making new friends | 2.05 | 2.01 | 2.06 | 2.03 | 2.06 | 2.03 |
| Maintaining friendships | 1.95 | 2.04 | 1.96 | 2.10 | 1.96 | 2.10 |
| Networking with the “right” people | 2.15 | 1.97 | 2.19 | 2.02 | 2.19 | 2.02 |
| Feeling pressured to socialize | 2.10 | 2.06 | 2.11 | 2.07 | 2.11 | 2.07 |
| Balancing a social life with academics | 2.52 | 2.55 | 2.41 | 2.44 | 2.41 | 2.44 |
| Comparing myself to others | 2.62 | 2.60 | 2.61 | 2.57 | 2.61 | 2.57 |
| Comparing myself to others on social media | 2.21 | 2.19 | 2.21 | 2.18 | 2.21 | 2.18 |
| Meeting others peoples’ expectations of me | 2.54 | 2.51 | 2.51 | 2.46 | 2.51 | 2.46 |
| Meeting my own expectations | 3.07 | 3.10 | 3.00 | 3.02 | 3.00 | 3.02 |
| ***Personal Domain*** |  |  |  |  |  |  |
| Making sure that I get enough sleep | 2.31 | 2.57 | 2.27 | 2.39 | 2.27 | 2.39 |
| Making sure that I get enough exercise | 2.35 | 2.55 | 2.32 | 2.39 | 2.32 | 2.39 |
| Making sure that I eat healthy | 2.27 | 2.51 | 2.26 | 2.34 | 2.26 | 2.34 |
| Having to prepare meals for myself | 1.95 | 2.18 | 1.94 | 1.88 | 1.94 | 1.88 |
| Balancing working at my job with academics | 2.73 | 2.73 | 2.63 | 1.77 | 2.63 | 1.77 |
| Balancing my extracurriculars with academics | 2.22 | 1.65 | 2.17 | 1.58 | 2.17 | 1.58 |
| Feeling guilty about taking time for my hobbies/interests | 2.51 | 2.48 | 2.51 | 2.34 | 2.51 | 2.34 |
| Having to take student loans | 1.42 | 1.30 | 2.73 | 1.24 | 2.73 | 1.24 |
| Worrying about paying off debt | 1.71 | 1.56 | 2.80 | 1.55 | 2.80 | 1.55 |
| Worrying about getting a job after graduating | 2.59 | 2.35 | 2.88 | 2.42 | 2.88 | 2.42 |
| Worrying about getting into a new program after graduating | 1.96 | 1.80 | 2.71 | 1.74 | 2.71 | 1.74 |
| Worrying about missing major “life events”  (i.e., buying a house, marriage, children) | 2.46 | 2.23 | 2.47 | 2.25 | 2.47 | 2.25 |
